# Previous Multiple Abdominal Surgeries: A Valid Contraindication to Abdominal Free Flap Breast Reconstruction?

**Published:** 2012-07-23

**Authors:** Michele Di Candia, Ahmed Al Asfoor, Zita M. Jessop, Devor Kumiponjera, Frank Hsieh, Charles M. Malata

**Affiliations:** ^a^Department of Plastic and Reconstructive Surgery; ^b^Clinical School of Medicine, Cambridge University; ^c^Cambridge Breast Unit, Addenbrooke's University Hospital, Cambridge University Hospitals NHS Foundation Trust, Cambridge, UK.

## Abstract

Presented in part at the following Academic Meetings:

- 57th Meeting of the Italian Society of Plastic, Reconstructive and Aesthetic Surgery, September 24-27, 2008, Naples, Italy.
- 45th Congress of the European Society for Surgical Research (ESSR), June 9-12, 2010, Geneva, Switzerland.
- British Association of Plastic Reconstructive and Aesthetic Surgeons Summer Scientific Meeting, June 30-July 2, 2010, Sheffield Hallam University, Sheffield, UK.

**Background:** Patients with previous multiple abdominal surgeries are often denied abdominal free flap breast reconstruction because of concerns about flap viability and abdominal wall integrity. We therefore studied their flap and donor site outcomes and compared them to patients with no previous abdominal surgery to find out whether this is a valid contraindication to the use of abdominal tissue. **Patients and Methods:** Twenty patients with multiple previous abdominal operations who underwent abdominal free flap breast reconstruction by a single surgeon (C.M.M., 2000-2009) were identified and retrospectively compared with a cohort of similar patients without previous abdominal surgery (sequential allocation control group, n = 20). **Results:** The index and control groups were comparable in age, body mass index, comorbidities, previous chemotherapy, and RT exposure. The index patients had a mean age of 54 years (r, 42-63) and an average body mass index of 27.5 kg/m^2^ (r, 22-38). The main previous surgeries were Caesarean sections (19), hysterectomies (8), and cholecystectomies (6). They underwent immediate (n = 9) or delayed (n = 11) reconstructions either unilaterally (n = 18) or bilaterally (n = 2) and comprising 9 muscle-sparing free transverse rectus abdominis muscle and 13 deep inferior epigastric perforator flaps. All flaps were successful, and there were no significant differences in flap and donor site outcomes between the 2 groups after an average follow up of 26 months (r, 10-36). **Conclusion:** Multiple previous abdominal surgeries did not predispose to increased flap or donor site morbidity. On the basis of our experience, we have proposed some recommendations for successful abdominal free flap breast reconstruction in patients with previous multiple scars. Careful preoperative planning and the use of some intraoperative adaptations can allow abdominal free flap breast reconstruction to be reliably undertaken in such patients.

Use of abdominal tissue is considered the best available option for autologous breast reconstruction after mastectomy. Patients with significant abdominal scars from previous surgeries frequently present for breast reconstruction and often seek or are only suited for autologous tissue reconstruction. The use of pedicled and free abdominal flaps in such patients with multiple preexisting abdominal scars has many potential problems.^1^ The vascularity of part or whole of the flap in these patients is potentially unreliable thus precluding its use as the first choice for many.^2^^-^4 This is compounded by the concerns regarding surgical disruption of the integrity of the abdominal wall which may lead to weakness, laxity, bulges, or frank herniation. Various operative strategies have been suggested to improve transverse rectus abdominis muscle (TRAM) flap survival and reduce donor site morbidity in patients with preexisting abdominal scars with variable success.^3^^-^9

Preexisting single abdominal scars have been shown not to increase the flap complications in patients undergoing abdominal flap breast reconstruction^5^^,^^7^^,^^9^ but may increase donor site morbidity.^5^^,^^10^^,^^11^ Previous multiple abdominal surgeries have been documented to increase postoperative abdominal wall morbidity following abdominal flap harvest but not shown to increase the risk of flap failure.^3^^-^8

There are numerous studies looking into the effect of single abdominal scars on flap and donor site outcomes,^3^^,^^4^^,^^6^^,^^9^^,^^12^^-^14 in contrast to the paucity of literature concerning the effect of previous multiple abdominal surgeries.^8^ In our practice, previous abdominal surgery is not considered to be a contraindication to abdominal flap breast reconstruction.^9^ Although patients may have a particular scar, it is more important to ascertain what surgery led to the scar because with each surgery there is increased danger to the vascularity with more scarring underneath. Depending on the locations of the scars and the type of previous surgery, the senior author (C.M.M.) makes adaptations to the reconstructive approach employed. The objective of this study was to compare the flap and donor site outcomes in patients with previous multiple abdominal surgery, who underwent an adapted approach to abdominal flap breast reconstruction, to those without previous abdominal surgery. The information gleaned could then be used in counseling patients with multiple previous abdominal surgeries requesting free flap breast reconstruction. It would also provide recommendations for successful execution of this surgery in this challenging group of patients.

## PATIENTS AND METHODS

Patients with previous multiple abdominal operations who underwent abdominal flap breast reconstruction by a single surgeon (C.M.M.) from January 2000 to December 2009 were identified from the free flap database. Their case notes were reviewed for data on indications for previous abdominal surgery and locations of the previous incisions (thus current scars).

The flap type (deep inferior epigastric perforator [DIEP], muscle-sparing [MS]-I, MS-II, MS-III TRAM),^10^ abdominal flap design, reconstruction timing (immediate or delayed), flap outcomes, and donor site morbidity were also recorded. A note was also made of patient's age, body mass index (BMI), smoking history, and comorbidities.

They were then retrospectively compared to a group of patients who underwent the same reconstruction but with no previous abdominal surgery. We used 2 control group designs in this study—one randomized (n = 20) and the other alternate sequential allocation (n = 20). The latter group was selected by choosing the patient immediately following each patient with multiple previous abdominal surgeries. The primary reason for these 2 control groups was to avoid selection bias and nullify the surgeon's inevitable learning curve with time (time-experience bias). Only patients operated on by the same surgeon (C.M.M.) were included to avoid interoperator variability.

We used total flap loss, partial flap loss, fat necrosis (subcutaneous firmness of at least 2 cm),^15^ wound infection, and wound breakdown as measures of flap outcomes. Donor site outcomes included seroma, wound infections (culture-proven or cellulitis needing antibiotics), wound breakdown, skin flap necrosis, and abdominal wall laxity (bulge, hernia, or weakness). Numerical data (age, BMI) were compared using Mann Whitney *U* test while categorical (nominal) data were compared with Fisher exact test.

Following flap harvest, the rectus muscle was approximated with a running 2/0 Vicryl suture while the anterior rectus sheath was repaired with running looped “0” Ethilon in 2 layers. None of the patients required mesh repair, and the abdominal wound closure was achieved with interrupted 2/0 PDS (Ethicon) to Scarpa's fascia and 3/0 Monocryl (Ethicon) in 2 layers (interrupted deep dermal and continuous subcuticular). Two suction drains were used and removed prior to discharge from hospital when draining less than 30 mL per day. A supportive binder was worn day and night for 3 months.

## RESULTS

Over the 10-year period, 20 patients with multiple previous major abdominal surgeries underwent unilateral (n = 18) or bilateral (n = 2) abdominal flap breast reconstruction (total = 22 flaps). There were 9 immediate and 11 delayed reconstructions. The reconstruction types included 9 free muscle-sparing TRAMs and 13 free DIEP flaps. Patients' ages ranged from 42 to 63 years (mean = 54) and the average BMI was 27.45 kg/m^2^ (r, 24-35). Three patients were smokers at the time of the surgery (15%) (Table 1). Fourteen patients had previous chemotherapy, and 10 had previous radiotherapy (RT). One patient had a history of a deep venous thrombosis (DVT) while another had a previous history of lymphoma. The abdominal scars encountered were Pfannenstiel, caesarean sections, midline and paramedian laparotomy, subcostal scars, nephrectomy, hernia repair, laparoscopic trocar sites, and appendicectomy (Table 2). The indications for the incisions were largely obstetric and gynecological. Most patients in the series had 2 scars but 2 patients had 5 abdominal scars each (Table 3). No previously damaged donor blood vessels were encountered in any of the patients at surgery. For the 13 DIEP flaps, 2 perforators were included in each flap; no flap was raised on a single perforator.

The breasts reconstructed were of the following cup sizes: C = 3, D = 7, and DD = 5 (unspecified = 5). The mastectomy specimens weighed an average of 675 g (r, 485-903). Five patients (38%) had contralateral balancing breast surgery comprising 3 breast reductions and 2 mastopexies.

There were no flap failures, and none of the flaps developed partial skin loss, infection, or significant fat necrosis. Five patients had donor site seroma, which needed aspiration, but none required operative drainage as they all settled with repeated aspirations (average = 3). All healing problems were minor and required no operative intervention. They comprised 3 partial wound breakdowns and 2 wound infections needing antibiotic treatment (Tables 4 and 5). One of the dehisced wounds required debridement and direct closure, as the patient was about to go on vacation and could not wait for spontaneous healing with conservative measures. After an average follow-up of 26 months (r, 10-36), none of the patients developed abdominal wall weakness, bulging, or frank herniation. One patient in the multiple scar group has noticed a localized bulge at the site of the DIEP flap harvest site but did not need treatment.

**Figure 1:**
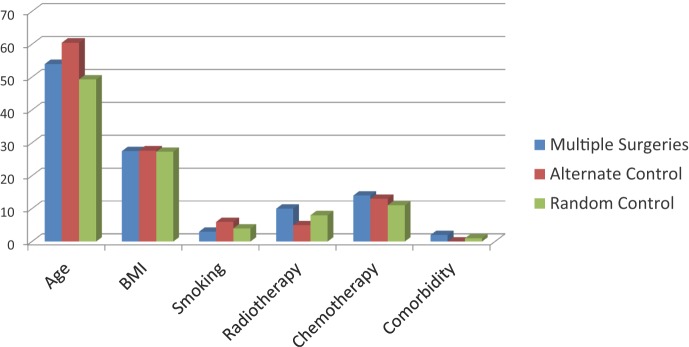
Baseline characteristics for index and control groups.

The index and 2 control groups were comparable in terms of age, BMI, previous cancer treatment, and comorbidities (Fig 1). There were no significant differences in flap and donor site outcomes between the patients with multiple previous abdominal scars and the controls groups (the results are shown for the sequential allocation control versus the index group in Tables 4 and 5, as these were identical when compared with the random controls)

## DISCUSSION

Our study showed that similar results can be obtained in patients who have had previous multiple abdominal surgeries, as in patients with no scars. This has been previously documented for patients with multiple scars. However, none of these previous studies have analyzed the effect of the previous multiple abdominal surgeries with respect to the indication for those surgeries. It is important to do this analysis because clinically at each operation the vessels (pedicle, perforators or both) are put at risk by the abdominal surgery even though it is being performed via the same incision. Therefore, an analysis of flap and donor site outcomes merely based on scars is an oversimplification.

Patients with previous major abdominal surgery are often informed that they are not suitable for abdominal flap breast reconstruction and in our institution often ended up in the senior author's clinic (C.M.M.) because he does not consider the scars of previous major abdominal surgery to be an absolute contraindication for surgery. Indeed, in his practice, almost a third of all patients who undergo abdominal flap breast reconstruction have had preexisting scars.^9^

**Figure 2:**
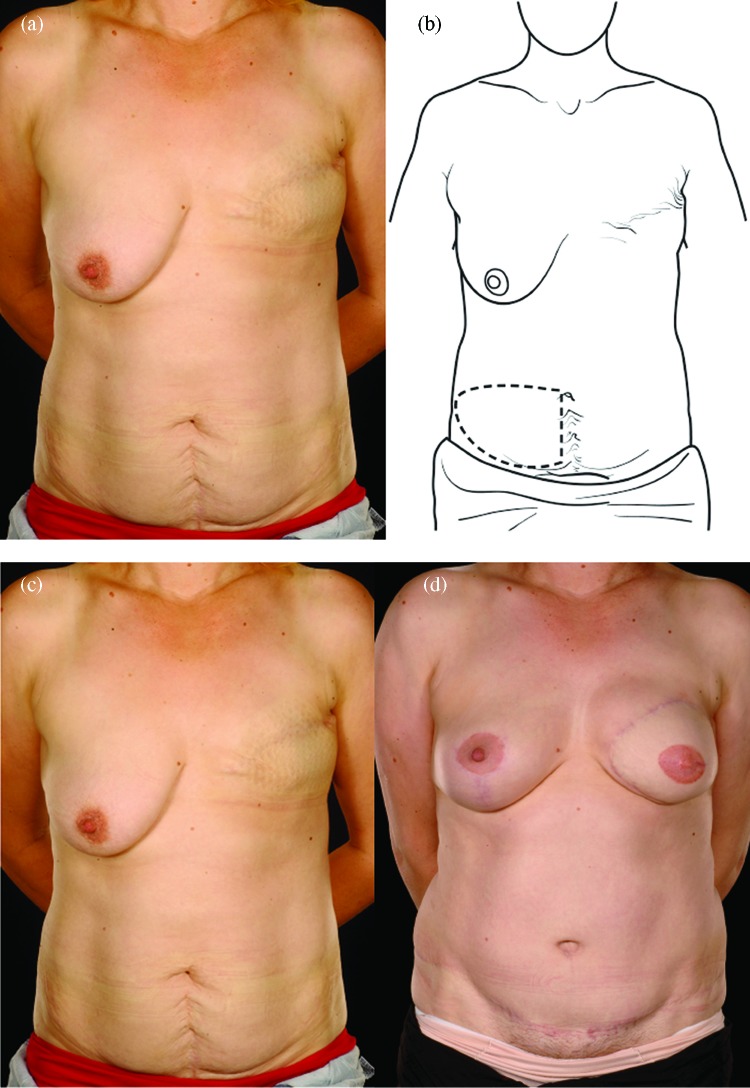

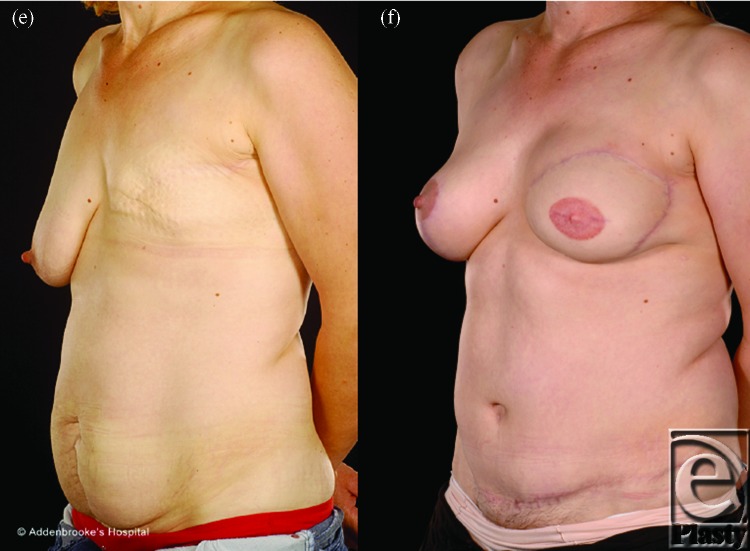
Two abdominal scars: A 30-year-old lady with 2 scars, one from a classical emergency Caesarian section and the other from a Pfannenstiel incision for an ectopic pregnancy presented for delayed breast reconstruction 2 years following a mastectomy performed outside the United Kingdom (*a*, *c*, *e*). She underwent a right hemi MS-II free TRAM flap (*b*). Half of her lower abdomen was sufficient to reconstruct her relatively small breast whose shape was made favorable by the simultaneous right LeJour mastopexy (*d*, *f*). MS indicates muscle sparing; TRAM, transverses rectus abdominis muscle.

**Figure 3:**
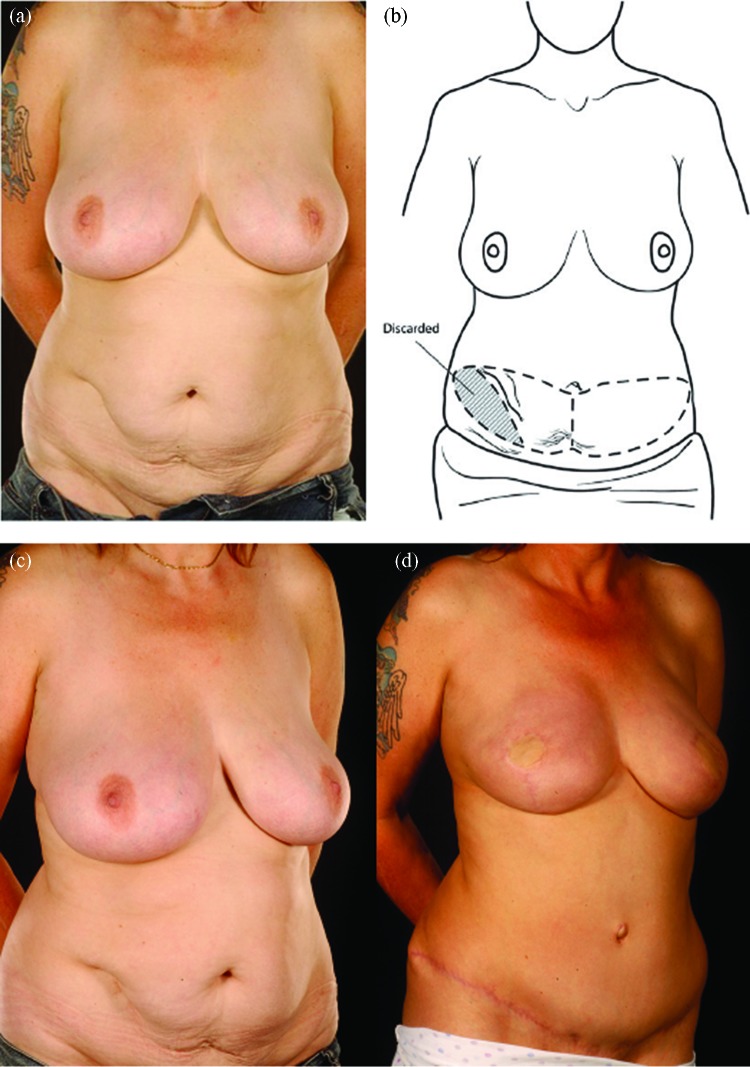

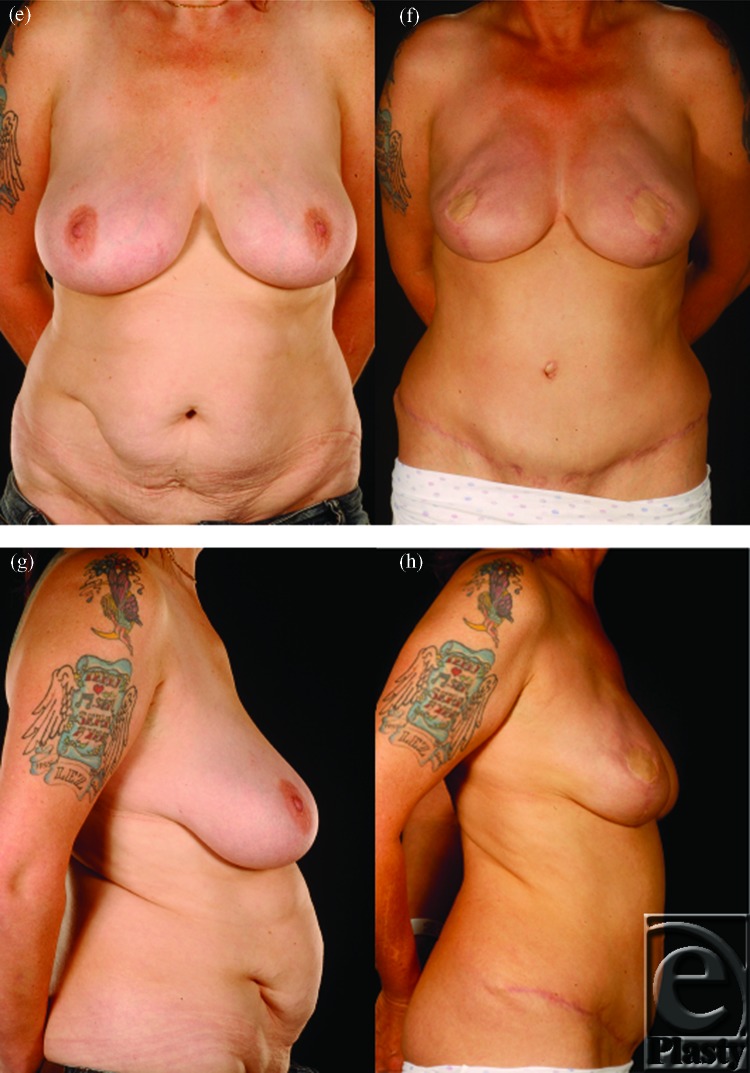
Five abdominal scars: A 36-year-old lady with 5 scars from hysterectomy, oophorectomy, appendicectomy, and 2 previous laparoscopies presented for bilateral prophylactic mastectomy (the last mentioned category of scars were counted as 2 scars as it was still possible to damage the donor vessels during laparoscopic procedures). (*a*-*c*) She was reconstructed with a right hemi free MS-II TRAM flap for the left breast and a left hemi DIEP for the right breast. Note the improved contour of her abdomen (*d*-*h*). MS indicates muscle sparing; TRAM, transverses rectus abdominis muscle.

**Figure 4:**
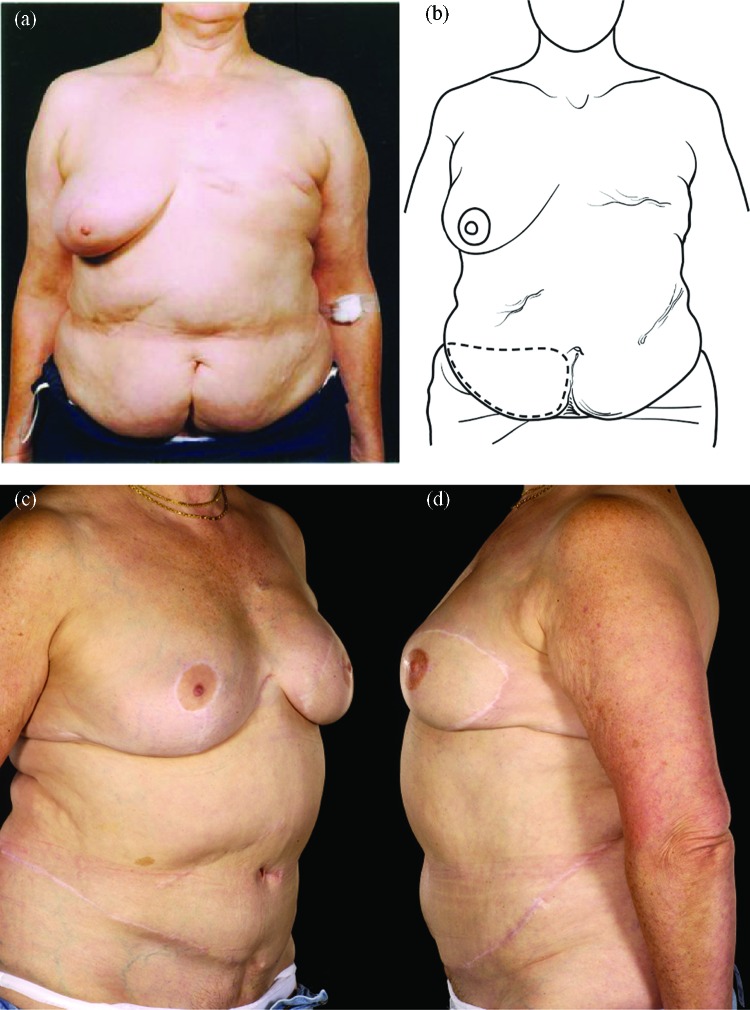

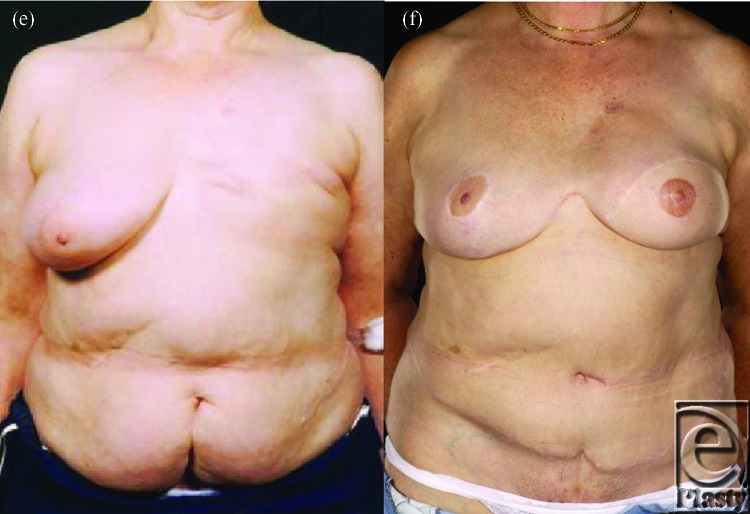
Four abdominal scars: A 59-year-old patient with 4 scars (Pfannenstiel, midline, right subcostal, and left nephrectomy) (*a*-*b*) underwent a skewed and extended right MS-II free hemi TRAM flap breast reconstruction (reproduced with permission from Hsieh et al^9^) achieved with minimal abdominoplasty flap undermining resulting in an eccentric transverse donor site scar. To obtain adequate volume, the larger right hemiflap was selected and harvested in an extended and skewed fashion. The final aesthetic outcome 8 months following a contralateral balancing superior pedicle breast reconstruction was satisfactory (*c*-*f*. MS indicates muscle sparing; TRAM, transverses rectus abdominis muscle.

**Figure 5:**
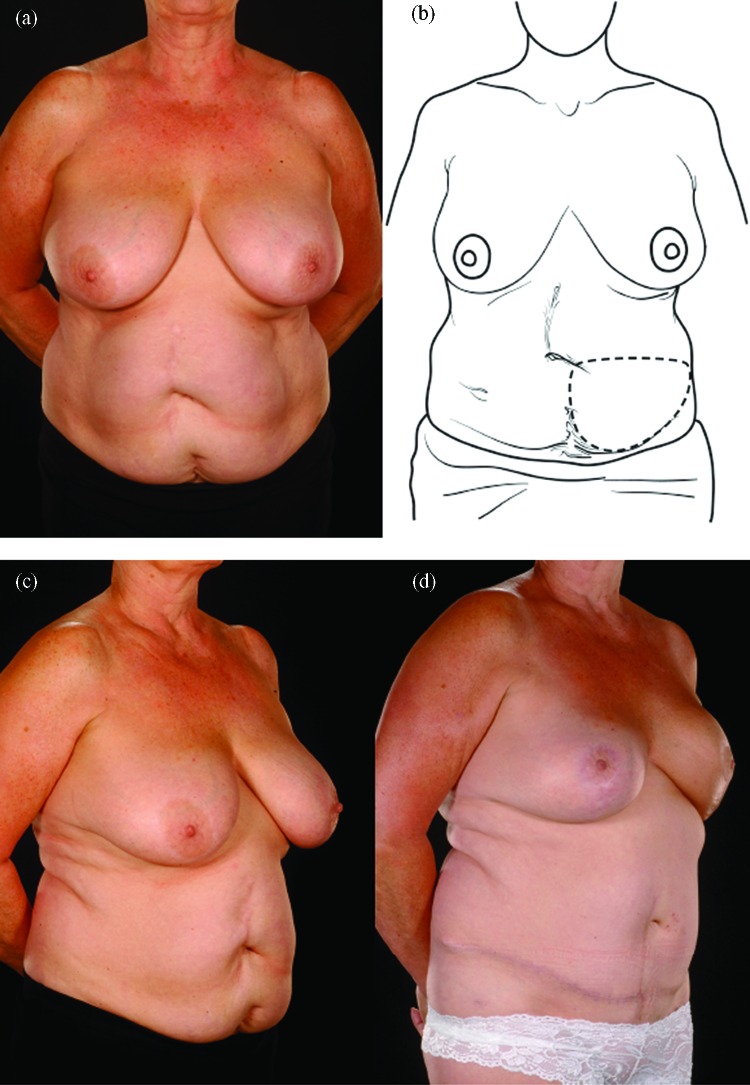

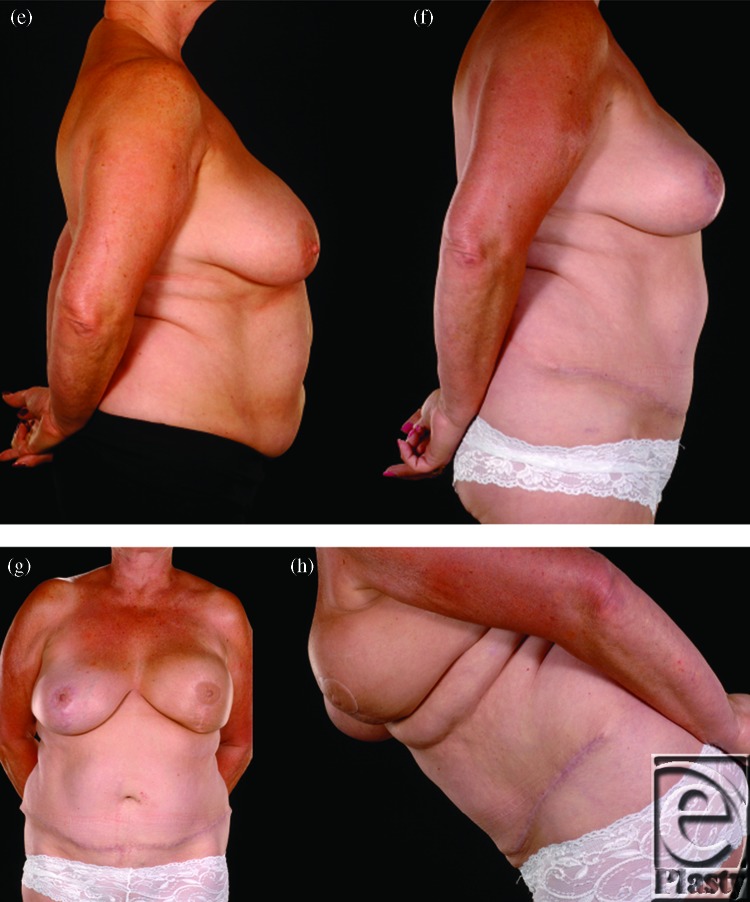
Five abdominal scars: A 61-year-old lady with 5 scars (grid-iron appendicectomy, 2 previous laparoscopies, Caesarian, and Pfannenstiel) (*a* and *b*) underwent a left immediate breast reconstruction with a left MS-I TRAM and a simultaneous contralateral balancing LeJour mastopexy. The appearance after 8 months shows that the reconstructed breast has shrunk following radiotherapy and has a small persistent inferior dog ear (*d*). The abdominal contour was improved by repairing of the rectus sheaths without recourse to the use of mesh (*d*-*g*). A diver's view confirms an intact abdominal wall without bulging or herniation (*h*). MS indicates muscle sparing; TRAM, transverses rectus abdominis muscle.

Reconstructing patients with previous multiple surgical scars through the abdominal wall is especially challenging because tissue availability is limited, flap vascularity is potentially unreliable, and flap harvest may weaken the abdominal wall (Fig 2). However, a number of these patients insisted on abdominal flap reconstruction despite these adverse conditions and potential problems (Figs 3-5). It is therefore important to document the outcomes of these reconstructions in comparison to patients with no previous abdominal surgery both in the immediate and delayed scenarios and “formulate” proposals for successful execution of this surgery.

**Figure 6:**
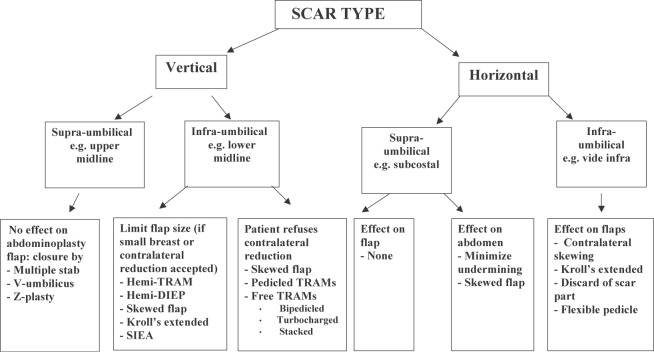
Algorithmic approach.

In our practice, different operative strategies have been adopted to overcome the limitations posed by abdominal scars especially midline and subcostal ones. The experience has led to formulation of an algorithmic approach to this problem^9^ (Fig 6). The precautions that have to be taken for successful abdominal flap breast reconstruction in patients with multiple previous abdominal surgery scars are summarized in Table 6.

### Flap types “multiple scars = a free flap”

Our series of multiple scar patients who had previously undergone multiple abdominal surgeries was nearly equally divided between immediate (9/20) and delayed (11/20) breast reconstructions. The most notable finding was that all patients with multiple scars were reconstructed with a free tissue transfer. This is in contrast with our overall experience with abdominal scars in which some flaps were pedicled.^9^ It is a reflection of the fact that free tissue transfers have a more robust blood supply compared to pedicled TRAMs.^16^^-^19 Furthermore, previous scarring may impair or compromise perforator anatomy or size. In addition to disturbing the perforators, abdominal scars may compromise or reduce the perfusion across the scar. We would therefore recommend that if possible more than one perforator should be used and if the scarring precludes perforator flap harvest then one should have a low threshold for intraoperative change of plan for free TRAM flap design.

### Surgical incision types

Although some authors contend that Pfannenstiel incisions should not affect the flap perfusion,^7^ this is by no means universally accepted. The effect of Pfannenstiel incisions on flap circulation depends on how extensive the subfascial and suprafascial dissection was during the surgery, the indication for this surgery and whether any complications (eg, hematoma or infection) occurred following surgery. The latter two may increase scarring thereby making dissection of the perforators difficult while extensive dissection could have damaged the perforators profoundly or irreversibly.

In patients with lower midline scars, only a small amount of tissue on the contralateral side can be reliably included in the flap, as this tissue is variably perfused depending on how old the scar is, its length, and its previous indication (Fig 5).

We therefore determine the perfusion of the contralateral tissue after vessel anastomoses and then discard the portions that are not well perfused. This is similar to the practice of others^8^ and provides a valuable increase in the horizontal dimension of the flap.

### Flap and donor site complications

Although Mehrara et al^20^ have documented increased complications of both the donor and flap sites, most authors would agree that preexisting abdominal scars do not increase flap complications. This is confirmed by our past and present studies.^9^

Previous surgery through the abdominal wall weakens the abdominal fascia and/or muscles and therefore it is not surprising that they may lead to abdominal wall laxity.^5^^,^^10^ We did not find an increased rate of donor-site complications in patients with multiple abdominal scars. This contrasts with the findings of Parrett et al (2008), who found a significantly higher rate of abdominal donor-site complications in their DIEP flaps in patients with scars versus those with no scars (24% vs 6%). Subcostal scars are a well-documented cause of increased donor site breakdown in TRAM flaps.^5^^,^^8^^,^^13^ None of our open cholecystectomy patients, however, experienced wound breakdown or skin flap necrosis largely due to the algorithmic precautions we undertook.^9^

### Perforator anatomy and vascular imaging

Although usually only one good perforator is mandatory for flap survival especially with an adequate vein (1.5-mm diameter),^15^^,^^21^^,^^22^ we strive to use 2 or more perforators in patients with preexisting multiple abdominal scars because of concerns that the scars might have affected perfusion within the flap.

This review study was undertaken before adopting preoperative localization of the perforators with computed tomographic (CT) angiography^23^^,^^24^ by our unit. This is similar to other large series on this subject.^8^ All patients, whether they have scars or not, had the percutaneous location of their perforators marked out using a hand-held Doppler probe preoperatively.^21^ We have recently adopted CT angiography for all our patients. These newer imaging techniques for perforator flap anatomy may in the future facilitate the performance of breast reconstructions in patients with multiple scars.^23^^,^^25^

### Tips for successful abdominal flap reconstruction in patients with multiple scars (multiple previous abdominal surgery)

Abdominal scars should, however, not be approached with trepidation in patients seeking abdominal flap breast reconstruction. Patients should be counseled about the possibility of an increased risk of donor site complications even with DIEP flaps.^8^ The precautions we undertake are summarized in Table 6 and the algorithmic approach in Figure 6. When faced with a patient who has multiple scars on her abdomen, one has to keep an open mind as to the most feasible approach. In our opinion, pedicled flaps are contraindicated unless the patient has minor scars such as those from laparoscopy or appendectomy. We select the laterality of the flap based on the side of the lower abdomen, which has the least number of scars and is therefore likely to have the least disruption to its blood supply. In cases in which bilateral reconstruction is required, the side with the most scars should be a muscle-sparing free TRAM flap as opposed to a DIEP flap (Figs 4 and 5).

It is important not to commit to a pedicle until exploration of the deep inferior epigastric vessels (DIEVs) has shown them not only to be intact and in continuity but also to be of adequate calibre and unaffected by any previous scarring. If on one side the vessels are scarred or of poor quality, then the other side must be explored and the flap pedicle should be changed appropriately. It is therefore vital that no skin be de-epithelialized before flap transfer and successful microvascular anastomosis. The surgeon therefore needs to be versatile in the use of both the left and right DIEVs, the internal mammary, and the axillary recipient sites as well as different flap inset orientations dictated not only by the shape of the breast but also by the size, site of the flap harvested, and some of its portions that perfuse satisfactorily after microvascular anastomoses.

The next important consideration is the size of the flap. It is vital to make the flap from a multiply scarred abdomen as large as possible and discard portions of it only after perfusion has been established. If the flap size is rather small, the patient should have been prepared or counseled and consented for contralateral balancing breast reduction or mastopexy. Hence, one has to use simultaneous contralateral balancing surgery liberally. Alternatively, fat transfer may be planned for a later stage to increase the size of the flap.

The donor site morbidity can be reduced by minimizing the tension on the abdominoplasty flap, minimal undermining in patients with transverse upper abdominal scars^6^^,^^9^^,^^13^ and appropriate management of the residual upper abdominal scars in the abdomen. The latter includes Z-plasties or multiple stab incisions of the vertical scars. Tension on the abdominoplasty flap can be reduced by minimizing the size of the skin paddle size and undermining of the mons pubis or inguinal areas as suggested by our algorithmic approach.^9^

This inevitably results in a high abdominoplasty scar, and the patient must be warned about this. This situation also pertains when the suprapubic area has been multiply scarred by multilevel incisions rendering it unusable for inclusion in the flap.

Although we prefer to use DIEP flaps whenever possible to reduce donor site morbidity,^22^^,^^26^^,^^27^ it is sometimes necessary to undertake intraoperative conversion to a free TRAM flap to improve the vascularity.^21^ Taken to its logical conclusion in patients with multiple scars, especially in the lower abdomen, if the DIEP vessels are not suitable or seem inadequate, one should convert the free tissue transfer to a pedicled flap albeit with a smaller tissue volume. We did not need to carry this out in any of our patients. Interestingly, although superficial inferior epigastric artery (SIEA) flaps have the least morbidity of all abdominal flap types,^2^ we did not use any for multiple scars because of their proneness to disruption of the superficial vessels and the variability/unreliability of the arterial supply.

Lower vertical and transverse scars of the lower abdomen were the commonest combination of 2 scars. We approach this relatively more common scenario by using a hemi-flap either as a DIEP or as a free muscle-sparing TRAM flap (Fig 2).^9^^,^^11^^,^^12^

Since the present study, we have adopted CT angiographic imaging to delineate perforator anatomy in terms of location and relative sizes. We hope that this will prove useful in optimizing the execution of breast reconstruction in patients with multiple previous abdominal surgeries/scars and render them more suitable for abdominal flap breast reconstruction.

## CONCLUSION

The results from this small single operator case series suggest that the presence of multiple abdominal scars following major abdominal surgery does not constitute an absolute contraindication to abdominal flap breast reconstruction. With careful preoperative planning and taking into account the appropriate precautions, it is possible to safely undertake abdominal flap breast reconstruction in this challenging group of patients. However, patients must be informed of the potential risks, especially to the donor site. It is concluded that abdominal free flap breast reconstruction is not contraindicated in patients with previous multiple abdominal surgery in which the vascular pedicle was still preserved.

## Figures and Tables

**Table 1:** Patients' characteristics: multiple abdominal surgeries (index) group compared with alternate allocation control group

	Index	Control
Age	54.06 average	60.4
BMI	27.5 average	27.7
Uni DIEP	11	14
Bil DIEP	2	1
TRAM	9	5
Delayed	11	7
Immediate	9	13
Smokers	3 (%)	6
Radiotherapy	10	5
Chemotherapy	14	13
Comorbidity	2*	None

*1 DVT and 1 lymphoma. DIEP indicates deep inferior epigastric perforator; DVT, deep venous thrombosis; TRAM, transverses rectus abdominis muscle.

**Table 2:** Abdominal scar types and their frequency in patients undergoing abdominal flap breast reconstruction

Scar Type	Patients (n = 20)
Caesarian section	19
Hysterectomy ± oophorectomy	6
Pfannenstiel	2
Appendicectomy	4
Hernia repair	2
Midline laparotomy	3
Paramedian laparotomy	2
Subcostal	2
Nephrectomy	2
Laparoscopy	3
Others	4

**Table 3:** Frequency of multiple abdominal scars in patients undergoing free flap breast reconstruction

Scar Group	Patients (n = 20)
Two scars	10
Three scars	4
Four scars	2
Five scars	2
Six Scars	2

**Table 4:** Donor site complications in patients with multiple abdominal scars versus alternate controls (index versus sequential allocation groups)

Donor Site Complications	Scar Group	Alternate Controls
Seroma required aspiration	5	6
Hernia/Bulge	1 bulge	0
Wound dehiscence	1	0
Infection	1	0
Total	5	6

**Table 5:** Flap complications in patients with multiple abdominal scars versus alternate controls (index versus sequential allocation groups)

Flap Complications	Scar Group	Alternate Controls
T junction dehiscence	2	1
Wound dehiscence	1	0
Infection	2	1
Hematoma	0	1
Total	5	3

**Table 6:** Precautions in abdominal flap breast reconstruction in patients with multiple preexisting scars (recommendations)

Principles
• Select a free tissue transfer in preference to pedicled flap if possible
• Restrict pedicled flaps to minor scars (laparoscopy or appendix)
• Explore the donor vessels first
• Do not commit to the side of donor vessels until the DIEVs have been visually demonstrated to be in continuity
• To be versatile in the use of left or right flap pedicles; internal mammary, axillary, and other recipient vessels; flap inset orientations: vertical, oblique, and horizontal
• Use contralateral balancing reductions or mastopexies liberally (as available flap tissue may be limited)
• Minimize abdominal donor site morbidity by limited abdominoplasty undermining, accurate repair of fascial weaknesses
• Convert to pedicled flap if both donor vessels previously divided or encased in scar tissue
• CT or MRI angiography of the deep inferior epigastric vessels: perforator anatomy

CT indicates computed tomography; DIEVs, deep inferior epigastric vessels; MRI, magnetic resonance imaging.
